# Proof of Reliability Convergence to 1 at Rate of Spearman–Brown Formula for Random Test Forms and Irrespective of Item Pool Dimensionality

**DOI:** 10.1007/s11336-024-09956-7

**Published:** 2024-03-12

**Authors:** Jules L. Ellis, Klaas Sijtsma

**Affiliations:** 1 Faculty of Psychology, Open University of The Netherlands, Heerlen, The Netherlands; 2 Radboud University Nijmegen, Nijmegen, The Netherlands; 3 Tilburg University, Tilburg, The Netherlands

**Keywords:** reliability, Spearman–Brown prophecy formula, item sampling, parallel measures, convergence

## Abstract

It is shown that the psychometric test reliability, based on any true-score model with randomly sampled items and uncorrelated errors, converges to 1 as the test length goes to infinity, with probability 1, assuming some general regularity conditions. The asymptotic rate of convergence is given by the Spearman–Brown formula, and for this it is not needed that the items are parallel, or latent unidimensional, or even finite dimensional. Simulations with the 2-parameter logistic item response theory model reveal that the reliability of short multidimensional tests can be positively biased, meaning that applying the Spearman–Brown formula in these cases would lead to overprediction of the reliability that results from lengthening a test. However, test constructors of short tests generally aim for short tests that measure just one attribute, so that the bias problem may have little practical relevance. For short unidimensional tests under the 2-parameter logistic model reliability is almost unbiased, meaning that application of the Spearman–Brown formula in these cases of greater practical utility leads to predictions that are approximately unbiased.

What happens to the reliability of a test if items from an infinite pool are randomly added to the test? We will argue that under general circumstances, the reliability will go to 1, and that the rate of this is given by the Spearman–Brown formula (Brown, 1910; Spearman, 1910). This is different from the common belief that items or other test parts need to be parallel for the reliability to go to 1. The Spearman–Brown formula is derived traditionally from the assumption that items or test parts are parallel, but we show that it also works under more general conditions. For practical test construction, this result shows that it always makes sense to add items to increase reliability, a result that many researchers know from experience but without the fundamental theoretical support our result provides. Prior to presenting the new results, to fresh up memory, we briefly reiterate the theoretical basis of the Spearman–Brown formula.

A test or a questionnaire consists of items or test parts, such as testlets consisting of short literary texts and a small number of questions identical with each text, that together produce a test score. Often, the test score is the sum of the item scores or the testlet scores. Many methods exist to estimate the reliability of the test score (e.g., Guttman, 1945, Jackson & Agunwamba, 1977, Sijtsma & Van der Ark, 2020, Ten Berge & Zegers, 1978). The test constructor or the researcher using a test or questionnaire may believe the test length is either too low or too high. The cause of the former concern is usually that she expects measurement precision or statistical power is too low, and the cause of the second concern may be that the test uses too many items putting too much mental burden on young children, busy teachers, or clinical patients. A well-known strategy for increasing or decreasing reliability is to add items or delete items until a desired value is obtained.

Let *n* be the factor by which the number of items or test parts is increased ($n>1$) or decreased ($0<n<1$), then the (generalized) Spearman–Brown formula states that if a test with reliability $\rho _{1}$ is lengthened or shortened with factor *n*, the reliability of the new test will be

$$\begin{aligned} \rho _{n}=\frac{n\rho _{1}}{1+\left( n-1 \right) \rho _{1}} \end{aligned}$$

In the derivation of this formula, it is usually assumed that items are parallel (Lord and Novick, 1968), and it is often emphasized that the formula holds *only* for parallel items. We will show that it provides a close approximation much more generally.

If the items are parallel with $\rho _{1}>0$, then the Spearman–Brown formula implies that the reliability approaches 1 if $n\rightarrow \infty$. However, it is easily seen that reliability will much more generally approach 1 if the number of items increases. One argument for this is based on coefficient alpha (Guttman, 1945). Coefficient alpha is often regarded as a lower bound to the reliability (see Sijtsma & Pfadt, 2021, for a discussion). For a test consisting of *n* items with inter-item covariances $c_{ij}$ and item variances $v_{i}$, if we denote the mean off-diagonal inter-item covariance as $\bar{c}_{n}:=\sum \limits _{i=1}^n \sum \limits _{j=1, j\ne i}^n c_{ij} /n(n-1$) and the mean item variance as $\bar{v}_{n}:=\sum \limits _{i=1}^n v_{i} /n$, and their ratio as $\bar{\rho }_{n}:= \bar{c}_{n}/\bar{v}_{n}$, then coefficient alpha, denoted $\alpha _{n}$ and known as

$$\begin{aligned} \alpha _{n}=\frac{n}{n-1}\left( 1-\frac{\sum \nolimits _{i=1}^n v_{i}}{\sum \limits _{i=1}^n \sum \limits _{j=1}^n c_{ij} } \right) \end{aligned}$$

can be written as

$$\begin{aligned} \alpha _{n}=\frac{n\bar{\rho }_{n}}{1+(n-1)\bar{\rho }_{n}} \end{aligned}$$

(e.g., Warrens 2015, p. 130). The result has the structure of the Spearman–Brown formula. From this result it follows that, as $n\rightarrow \infty$, $\alpha _{n}$ approaches 1 if and only if $n\bar{\rho }_{n}\rightarrow \infty$, for which it is sufficient—but not necessary—that $\bar{\rho }_{n}$ remains greater than some $\varepsilon >0$ Furthermore, under random sampling of items, if we denote the expected off-diagonal inter-item covariance as $\bar{c}_{\infty }$ and the expected variance as $\bar{v}_{\infty }$, and if we define $\bar{\rho }_{\infty }:= \bar{c}_{\infty }/\bar{v}_{\infty }$, the law of large numbers suggests that if the second moment of the $v_{i}$s is finite and $\bar{\rho }_{\infty }>0$, then $\bar{\rho }_{n}\rightarrow \bar{\rho }_{\infty }>0$ and therefore $\alpha _{n}\rightarrow 1$.

The above argument is not compelling, however, because even if the items are sampled independently, the inter-item covariances are not independent samples, and therefore application of the ordinary law of large numbers is not allowed. Furthermore, even if $\alpha _{n}\rightarrow 1$, the true reliability of the test might approach 1 at a faster rate if $\alpha _{n}$ is merely a lower bound. Therefore, we provide a more formal proof. We start with briefly reviewing the Spearman–Brown formula in classical test theory and generalizability theory. Then we explicate our notation and assumptions and present a theorem and its proof. We discuss a simulation study that investigates the effects of dimensionality on accuracy of the Spearman–Brown predictions for short tests. Finally, we discuss the consequences of our theorem for practical test construction.

## The Spearman–Brown Formula in Classical Test Theory

Because it is central in this article, we reiterate the (generalized) Spearman–Brown formula,

$$\begin{aligned} \rho _{n}=\frac{n\rho _{1}}{1+\left( n-1 \right) \rho _{1}} \end{aligned}$$

Many psychometric textbooks discuss the Spearman–Brown formula as an aid to “prophesize” what the reliability of a test would be after changing its length (e.g., Allen & Yen, 1979, pp. 85–88; Furr & Bacharach, 2008, p. 127; Nunnally, 1978, p. 243; Reynolds & Livingston, 2012, p. 131; Webb et al., 2006). The formula has also been used in studying how the power of statistical tests depends on the test length (Feldt, 2011, p. 425; Ellis, 2013, p. 19). Many textbooks mention the limitation that the formula assumes that the test parts are parallel (e.g., Lord & Novick, 1968, p. 139), an assumption that is usually not realistic. Is it possible to relax this assumption while maintaining the usefulness of the Spearman–Brown formula? An obvious but trivial relaxation is that it is also sufficient if the test components are parallel up to an additive constant, since adding a constant to one component would affect neither $\rho _{1}$ nor $\rho _{n}$, but the question remains whether it is possible to relax this assumption further in a more meaningful way.

Lord (1955) and Cronbach et al. (1972) argued that the assumption of parallel items is not needed to estimate reliability if one assumes that the items are randomly drawn from a large pool. It can be argued that this is also true for the Spearman–Brown formula. This article investigates the matter from the perspective of the test constructor. We assume that the test constructor starts with a test of given length, for example 10 items, and that she knows the reliability of the test score. Suppose she wishes to predict what the reliability will be after lengthening the test length to 25 items, randomly drawn from the same pool. In this study, we ask under which circumstances the Spearman–Brown formula provides reliability estimates with no or little bias. Specifically, does the accuracy of the Spearman–Brown formula depend on the dimensionality of the items, as is suggested by the condition that the items need to be parallel, or is random sampling of items sufficient to guarantee estimation accuracy?

## The Spearman–Brown Formula in Generalizability Theory

In generalizability theory (Cronbach et al., 1972; Gleser et al., 1965; Webb et al., 2006), it is assumed that the items are randomly sampled, while the assumption of parallel test items is often considered unnecessary. For example, Rajaratnam et al. (1965, p. 40) discuss the concept of “randomly parallel tests,” which “are formed by drawing items randomly from the universe as a whole,” and they state, “For randomly parallel tests, $\alpha$ obeys the Spearman–Brown formula as $k^{'}/k (n/n'$; the authors) departs from 1.00” (p. 50). A version of the Spearman–Brown formula is often used implicitly in generalizability theory. For example, consider a one-facet design of items and denote the observed-score variance $\sigma _{n}^{2}$, universe-score variance $\tau ^{2}$, residual variance $\varepsilon _{n}^{2}$, and generalizability $\rho _{n}$ for a test of length *n*. Assuming $\sigma _{n}^{2}= \tau ^{2}+ \varepsilon _{n}^{2}$ and defining $\rho _{n}=\tau ^{2}/\sigma _{n}^{2}$, we have the following equivalence:

$$\begin{aligned} \text {The Spearman--Brown formula holds for}\, \rho _{n} \text { if and only if}\, \varepsilon _{n}^{2}=\varepsilon _{1}^{2}/n \end{aligned}$$

The identity $\varepsilon _{n}^{2}=\varepsilon _{1}^{2}/n$, or versions of it for more complex designs, is routinely assumed in studies on the optimization of generalizability (e.g., Marcoulides, 1993, Marcoulides, 1995, Marcoulides, 1997, Marcoulides & Goldstein 1990, 1992; Meyer et al., 2013; Peng et al., 2012, Sanders, 1992, Sanders et al., 1989, 1991, Woodward & Joe, 1973).

We focus primarily on reliability, not generalizability. To clarify the difference, consider the situation of a test of *n* items selected from an infinite pool. Let $X_{n}$ be the observed-score variable of the test, $T_{n}$ the associated true-score variable, and $T_{\infty }$ the universe-score variable, defined on the entire item pool. We study the correlation between $X_{n}$ and $T_{n}$ as *n* increases, whereas generalizability theory would study the correlation between $X_{n}$ and $T_{\infty }$. Obviously, these topics are closely related but not identical. The reliability coefficient may be estimated without reference to generalizability for example, in a confirmatory factor analysis framework, such as McDonalds omega (see Zinbarg et al., 2005; 2006) or in IRT models (e.g., Kim & Feldt, 2010). The question is what happens to this reliability if the number of items changes.

## Notation and General Assumptions

### Notation of Conditional Expectations

We use results from the measure-theoretical foundations of conditional expectation (e.g., Billingsley, 1986, Majerek et al., 2005), and to do so smoothly, we use the notation that is common in the theory of conditional expectations, which differs from the conventional notation in generalizability theory: If *X* and *Y* are random variables on a common probability space, then the conditional expectation of *X* given *Y* will be denoted as $\mathbb {E}(X\vert Y$). This is a random variable, a function of random variable *Y*, and for a specific value *y* this random variable assumes the value $\mathbb {E}(X\vert Y=y$).

### Definition of Item True scores and Item Error Scores

Assume that we have a set of test items denoted $\Omega$ where individual items are indicated by subscript $\omega \in \Omega$. Assume that each item $\omega \in \Omega$ has an observed-score random variable $X_{\omega }$ with finite nonzero variance, and that a joint distribution exists for the $\{X_{\omega }\vert \,\omega \in \Omega \}$. Let $\mathcal {F}$ be a collection of variables in the same probability space. We define

$$\begin{aligned} T_{\omega }:=\mathbb {E}(X_{\omega }\vert \mathcal {F}\mathbf {)} \end{aligned}$$

and $E_{\omega }:=X_{\omega }-T_{\omega }$ for each item $\omega \in \Omega$. Assuming these conditional expectations exist, this implies that $\mathbb {E}\left( E_{\omega } \right) =0$ and $\textrm{cov}\!\left( E_{\omega },T_{\nu } \right) =0$ for all $\omega , \upsilon \in \Omega$. Henceforth, the $T_{\omega }$ are called the true-score variables and the $E_{\omega }$ are called the error-score variables, but we do not assume that these variables necessarily have the interpretation that is often given to them in texts on classical test theory (e.g., Lord & Novick, 1968, chap. 2). For example, we do not say that $T_{\omega }$ should be defined by an infinite series of replications within subjects. We say that the error-score variables are *uncorrelated *if

$$\begin{aligned} \textrm{cov}\!\left( E_{\omega },E_{\nu } \right) =0 \ \text {for all}\ \omega , \upsilon \in \Omega , \omega \ne \upsilon \end{aligned}$$

We do not create a new concept of true scores but rather aim at a definition that allows maximal generality of our results. Our definition of true scores is very general: True scores are conditional expectations. This encompasses multiple true-score concepts that have been defined earlier in the literature. Some examples are the following:

1. Assume that the items satisfy a common factor model $X_{\omega }=\sum \limits _d \lambda _{\omega d} F_{d}+U_{\omega }$, where the $F_{d}$ are common factors, the $U_{\omega }$ are unique factors, and the $F_{d}$ and $U_{\omega }$ have a centered multivariate normal distribution with correlations 0. With $\mathcal {F}=(F_{1},F_{2},...$), we have $T_{\omega }=\sum \limits _d \lambda _{\omega d} F_{d}$ and $E_{\omega }=U_{\omega }$.
2. Assume that the items satisfy an item response theory model where the $X_{\omega }$ are conditionally independent given some latent variable vector $\varvec{\Theta }$. Note that we do not put any restriction on the dimensionality of $\varvec{\Theta }$. Following Dimitrov (2003), Ellis (2021), Holland and Hoskens (2003), Kim and Feldt (2010, p. 180), Lord (1980, p. 46), and Stout (1990), among others, we can use this point of departure to define “model-based” true-score variables $T_{\omega }=\mathbb {E}(X_{\omega }\vert \varvec{\Theta }\mathbf {)}$, and corresponding error-score variables $E_{\omega }=X_{\omega }-T_{\omega }$. This is a special case of the general definition if we set $\mathcal {F}=\varvec{\Theta }$. Zimmerman (1976) gave a comparable definition, albeit without explicit reference to a latent variable. The assumption that the $\{E_{\omega }\vert \omega \in \Omega \}$ are uncorrelated now follows from the assumption of “local independence” of item response theory.
3. If sampling of observed scores within persons is defined, as described in Lord and Novick (1968, Chapter 2), and *V* is a variable that indicates the persons, then let $\mathcal {F}=V$ and hence $T_{\omega }=\mathbb {E}(X_{\omega }\vert V)$, and $E_{\omega }=X_{\omega }-T_{\omega }$. Lord and Novick’s assumption of “linear experimental independence” now implies that the error-score variables are uncorrelated.
4. Assume that the items satisfy a linear or nonlinear regression model with a set of predictors $\textbf{U}$. If we set $\mathcal {F}=\textbf{U}$, the predicted scores are $T_{\omega }=\mathbb {E}(X_{\omega } \vert \textbf{U})$, and the residuals are $E_{\omega }=X_{\omega } -T_{\omega }$. The assumption of uncorrelated errors now corresponds to uncorrelated residuals.
5. Ellis and Junker (1997) and Junker and Ellis (1997) argue that one can define “tail-conditional” true-score variables $T_{\omega }=\mathbb {E}(X_{\omega }\vert \varvec{\tau }(\textbf{X}))$, where $\varvec{\tau } (\textbf{X})$ is the tail sigma-field of the observed-score variables. This corresponds to $\mathcal {F}=\varvec{\tau } (\textbf{X})$.

Assume that the variables $X_{\omega }, T_{\omega }$, and $E_{\omega }$ have finite and positive variance. Let the standard deviations of $X_{\omega }, T_{\omega }$, and $E_{\omega }$ be $\sigma \!\left( \omega \right) \!,\tau (\omega$) and $\varepsilon (\omega$), respectively.

### Assumption of Random Selection of Items

Like Hunter’s (1968) probabilistic foundation of generalizability theory, we assume that some probability space is defined for $\Omega$, which means that the items can be drawn randomly. Let $\varvec{\Gamma }$ be the common sample space upon which the $X_{\omega }$s are defined; then henceforth we use the product probability space of $\Omega \times \Gamma$. This means that after drawing an item $\omega \in \Omega$, we can observe $X_{\omega }$. Let $R_{1}, R_{2},...$ be an infinite sequence of independent identically distributed (i.i.d.) random variables with range in $\Omega$. Here, $R_{i}$ is supposed to be the name or number of the *i*-th item during the random selection. Consistent with large parts of generalizability theory (e.g., Cronbach et al., 1972), we assume in the sequel that the item pool is infinitely large and that it is almost impossible that the same the item is included twice, that is, $P\!\left( R_{i}=R_{j} \right) =0$ for $i\ne j;\,i,j\in \mathbb {N}$. We furthermore assume that the $R_{1}, R_{2},...$ are independent of $\mathcal {F}, X_{\omega }, T_{\omega }$, and $E_{\omega }$ for all $\omega \in \Omega$.

### Assumptions on Moments

We assumed that $0< \textrm{var}(X_{\omega })<\infty$, $\textrm{var}(T_{\omega })<\infty$, and $\textrm{var}(E_{\omega }) <\infty$ for all $\omega \in \Omega$, and we furthermore assume that $\textrm{var}(\varepsilon ^{2}( R_{i} ))<\infty$ and $\textrm{var}(\tau ^{2}( R_{i} ))<\infty$ and $\vert \mathbb {E} (T_{R_{i}} )\vert <\infty$ and $\mathbb {E}( T_{R_{i}}^{2}) <\infty$ for all $i\in \mathbb {N}.$

### Definition of Test True Scores and Universe Scores

Test length is denoted by $n\in \mathbb {N}$. Let ${\textbf{S}_{n}:=(R}_{1}, R_{2},...,R_{n}$); this is a random vector, and each realization of it is a random test form of length *n*. The observed-score variables of the random test form are

$$\begin{aligned} X_{R_{1}}, X_{R_{2}},...,X_{R_{n}} \end{aligned}$$

These are different from the original observed-score variables in that the items are shuffled. For example, if $A,B\in \Omega$, then $X_{R_{1}}=X_{A}$ for some realizations, but $X_{R_{1}}=X_{B}$ for some other realizations. For random test form $\textbf{S}_{n}$, we define the test observed-score, true-score, and error-score variables as

$$\begin{aligned}{} & {} X_{\textbf{S}_{n}}:=\sum \limits _{i=1}^n {X_{R_{i}}/n} \\{} & {} T_{\textbf{S}_{n}}:=\sum \limits _{i=1}^n {T_{R_{i}}/n} \\{} & {} E_{\textbf{S}_{n}}:=\sum \limits _{i=1}^n {E_{R_{i}}/n} \end{aligned}$$

respectively. Since it is assumed that $|\mathbb {E}(T_{R_{i}})|<\infty$, we can define

$$\begin{aligned} T_{\infty }:=\mathbb {E}\left( T_{R_{1}}\,\vert \,\mathcal {F}\right) \end{aligned}$$

which may be called the universe-score variable.

### Definition of Reliability

We define the *reliability of test form* $\textbf{S}_{n}$ as

$$\begin{aligned} \rho \! \left( \textbf{S}_{n} \right) :=\frac{\mathrm{var\!} \left( T_{\textbf{S}_{n}}\,\vert \,\textbf{S}_{n}\right) }{\mathrm{var\!}\left( X_{\textbf{S}_{n}}\,\vert \, \textbf{S}_{n}\right) } \end{aligned}$$

For example, if the item pool is $\Omega =\left\{ \hbox {A}, \hbox {B}, \hbox {C},...\right\}$ and $n=3$ then one possible realization of the random test form is $\textbf{S}_{3} =\left( R_{1},R_{2},R_{3},\right) =\left( A, D,C \right)$ with reliability $\rho (\left( A, D,C \right)$), and another possible realization is $\textbf{S}_{3}=\left( R_{1},R_{2},R_{3}, \right) =(E,A,B$) with reliability $\rho ((E,A,B)$).

The reliability definition does not introduce a new concept of reliability; it simply denotes the population value of the reliability of total scores of a set of items $\textbf{S}_{n}$. In an earlier section we discussed different true-score variables, all special cases of our general definition, and the choice for a specific kind of true-score variables determines which estimation methods are appropriate. For example, if the true-score variables are defined by a linear factor model, $\rho \! \left( \textbf{S}_{n} \right)$ is the population value that can be estimated by McDonald’s $\omega _{t}$ (in the notation of Revelle and Zinbarg (2009)). In the same vein, if the true-score variables are defined on an item response theory model, $\rho \! \left( \textbf{S}_{n} \right)$ can be estimated as the “IRT reliability” described by Kim and Feldt (2010) and the “manifest reliability” described by Milanzi et al. (2015), also described by Dimitrov (2003). If the true scores are defined as within-subject expectations, and an experimentally independent retest with the same true-score variables exists, $\rho \! \left( \textbf{S}_{n} \right)$ can be estimated as the test–retest reliability of the test form $\textbf{S}_{n}$. As an aside, this is an important insight because psychometricians who estimate reliability with factor analysis tend to assume that a factor model is needed for classical test theory. It is not.

## Theorem on Reliabilities in Long Random Test Forms

In this section the effect of test length on reliability is studied theoretically. Note that for random test forms, at any length *n* there are multiple reliabilities, because $\rho \! \left( \textbf{S}_{n} \right)$ is a random variable. Nevertheless, we sometimes use the phrase “the reliability,” in singular, in informal texts. The question is whether reliabilities of random tests approach 1 as the test length increases, and whether the rate of convergence is given by the Spearman–Brown formula. For the latter question, we use the function

$$\begin{aligned} SB\! \left( x,n \right) :=\frac{nx}{1+\left( n-1 \right) x} \end{aligned}$$

For fixed *n*, the inverse function for *x* is ${SB}^{-1}\! \left( x,n \right) =SB\! \left( x,1/n \right)$, which is often implicitly used in calculations of lengthening or shortening the test with the Spearman–Brown formula. We will therefore study whether

$$\begin{aligned} SB\left( \rho (\textbf{S}_{n}),\frac{1}{n} \right) \ \text {converges to a real number for}\ n\rightarrow \infty \end{aligned}$$

If so, we may say that rate of convergence is given by the Spearman–Brown formula.

Recall that the section “Notation and general assumptions” described assumptions that hold throughout this article: randomly selected items, uncorrelated error-score variables, finite second moments. We now state the theorem that is our main result.

### Theorem

Assume that the error-score variables are uncorrelated and that the true-score variables are bounded by some square integrable random variable $T_{\textrm{max}}$, that is, $\left| T_{\omega } \right| <T_{\textrm{max}}$ for all $\omega \in \Omega$ and $\mathbb {E}\left| T_{\textrm{max}} \right| ^{2}<\infty$. For the reliabilities $\rho (\textbf{S}_{n}$), as $n\rightarrow \infty$ it holds that

$$\begin{aligned} SB\left( \rho (\textbf{S}_{n}),\frac{1}{n} \right) \rightarrow \frac{\mathrm{var\!}\left( T_{\infty } \right) }{\mathrm{var\!} \left( T_{\infty }\right) +\mathbb {E}(\varepsilon ^{2}\! \left( R_{1} \right) )} \end{aligned}$$

with probability 1.

The proof of the theorem is deferred to the appendix, but the basic idea of the proof is that $T_{{\textbf{S}}_{n}}\rightarrow T_{\infty }$ by the strong law of large numbers for conditional expectations (Majerek et al., 2005; Walk, 2008), and $\sum \limits _{i=1}^n {\varepsilon ^{2}\! \left( R_{i} \right) } /n\rightarrow \mathbb {E}(\varepsilon ^{2}\! \left( R_{1} \right)$) by the ordinary strong law of large numbers. Note that the convergence type in the theorem is specified as “with probability 1.” This means that the event of pointwise convergence has probability 1 (e.g., Billingsley, 1986, pp. 54, 290).

### Corollary 1

Under the conditions of Theorem 1, if furthermore $\mathrm{var\!}\left( T_{\infty } \right) >0$, then the reliabilities of the random test forms (i.e., the $\rho (\textbf{S}_{n}$)) converge to 1 with probability 1, and their rate of convergence is given by the Spearman–Brown formula.

Note that this result does not require that the items are unidimensional in any sense; it suffices to have uncorrelated error-score variables and randomly selected items, together with finiteness of the relevant moments. Finiteness of the relevant moments is assured if the observed-score variables are bounded, which is usually the case in real psychometric applications. Furthermore, this result pertains to “true” reliability. That is, it assumes that reliability is correctly estimated for each test length, and the theorem does not claim that a similar convergence would also hold for estimates such as Cronbach’s alpha, which may underestimate the true reliability (the theorem does not contradict it either).

Although reliability usually converges to 1, it is possible to create—rather artificial—examples in which reliability does not converge to 1:

1. If the conditions of Theorem 1 hold while $\mathrm{var\!}\left( T_{\infty } \right) =0$, then reliability converges to 0. The case of $\mathrm{var\!}\left( T_{\infty } \right) =0$, however, seems rather exceptional. For this to happen, we need that either all correlations between the observed-score variables are exactly 0 or that the positive and negative covariances cancel against each other exactly in the total item pool.
2. If, under the sampling of items, the error variances are equal to the square of a variable with a Cauchy distribution, then the sample mean of the error variances does not converge to 0. For essentially $\tau$-equivalent items, this implies that reliability does not converge to 1, since the sample mean of the true-score variables is constant. However, the Cauchy distribution is considered unrealistic since the time of its invention, especially for errors of measurement instruments (Stigler, 1974). Observed scores of items in psychometrics are usually on a scale with finite minimum and maximum, like 0 and 1, which excludes a Cauchy distribution.

## Simulation Studies

### Simulation Study 1: Unidimensional versus Multidimensional

In this section, we study how well the approximation established in the theorem holds in relatively short tests. To this end, Monte Carlo simulations generated item pools of 1000 items that satisfied the multidimensional 2-parameter logistic (2PL) model with up to five dimensions, $\varvec{\Theta }=(\Theta _{1},..., \Theta _{5}$) where each item loaded on precisely one dimension, denoted as $\textrm{dim}(\omega )$. The probability of a positive response is then

$$\begin{aligned} P\left( {X_{\omega }=1}\,\vert \,\varvec{\Theta }\right) =\frac{1}{1+\exp \left( -Da_{\omega }\left( \Theta _{\textrm{dim} \left( \omega \right) }-b_{\omega } \right) \right) } \end{aligned}$$

with $D=1.7$. The item parameters $a_{\omega }$ and $b_{\omega }$ were generated by a 4-parameter beta distribution with hyperparameters $\alpha , \beta$, minimum, and maximum. Note that the item parameters are written with Roman letters, $a_{\omega }$ and $b_{\omega }$, while the Greek letters $\alpha , \beta$ are parameters of the distribution where $a_{\omega }$ or $b_{\omega }$ is drawn from; that is, $\alpha$ and $\beta$ are characteristics of the entire item pool rather than of individual items. The item pools were designed with the following characteristics:

1. Number of dimensions. Item pools of 1, 2, or 5 dimensions were studied. Each item loaded on only one dimension. Each dimension in the pool was equally probably in the sense that each dimension was expected to be represented by approximately the same number of items.
2. Maximum discrimination parameter. The discrimination parameter $a_{\omega }$ was sampled from a beta distribution with minimum 0 and maximum either $a_{\textrm{max}}=2$ or $a_{\textrm{max}}=5$. The maximum 5 is rare in psychological tests but is sometimes obtained in healthcare applications (Hays et al., 2000, p. 4; Yang & Kao, 2014, p. 172).
3. Shape of the distribution of discrimination parameter. The $\alpha$ and $\beta$ hyperparameters of the beta distribution of the discrimination parameter $a_{\omega }$ were set such that the distribution was unimodal with $\alpha +\beta =12$ or (reverse) J-shaped with $\alpha +\beta =2$.
4. Mean discrimination parameter. The $\alpha$ and $\beta$ hyperparameters of the beta distribution of the discrimination parameter were set such that the mean of $a_{\omega }$ could be 0.83, 2.5, or 4.17 if $a_{\textrm{max}}=5$, or 0.33, 1.0, or 1.67 if $a_{\textrm{max}}=2$.
5. Mean difficulty parameter. The difficulty parameters $b_{\omega }$ were drawn from a beta distribution with minimum $-2$ and maximum 2, with a unimodal distribution ($\alpha +\beta =4$) having mean $-1$, 0, or 1.

For each value of the number of dimensions (1, 2, or 5) and each value of the maximum discrimination parameter (2 or 5) there were 18 “parameter cases” characterized by the distributions of the discrimination and difficulty parameters (6 possible distributions of $a_{\omega } \quad \times$ 3 possible distributions of $b_{\omega }$). For each parameter case, we generated an item pool of 1000 items and used this pool to generate 1000 random test forms of 50 items, in steps of 5 items. At each step the reliability was computed for each test form by numerical integration, assuming a standard normal distribution of the latent ability on each dimension, where the dimensions were independent of each other.

**Fig. 1:**
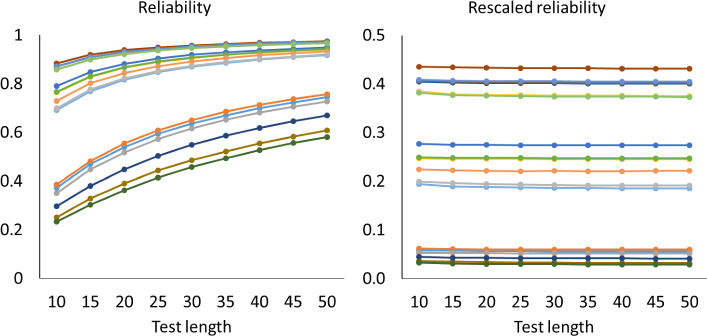
Mean reliabilities in Simulation Study 1 for unidimensional cases with $a_{\textrm{max}}=2$. Note. Mean reliability and mean rescaled reliability as a function of test length, in 18 cases of unidimensional models. The cases are represented by different colors. Each point is based on 1000 random test versions.

**Fig. 2:**
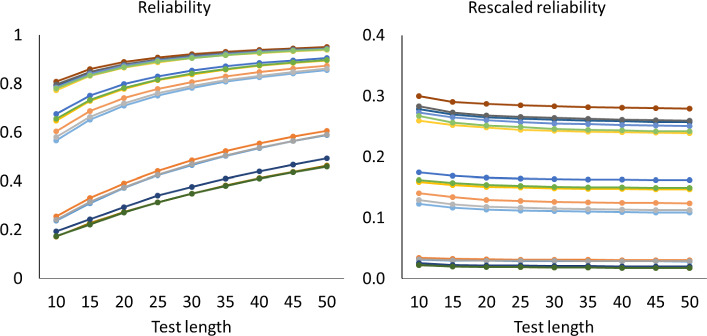
Mean reliabilities in Simulation Study 1 for two-dimensional cases with $a_{\textrm{max}}=2$. Note. Mean reliability and mean rescaled reliability as a function of test length, in 18 cases of two-dimensional models. The cases are represented by different colors. Each point is based on 1000 random test versions.

**Fig. 3:**
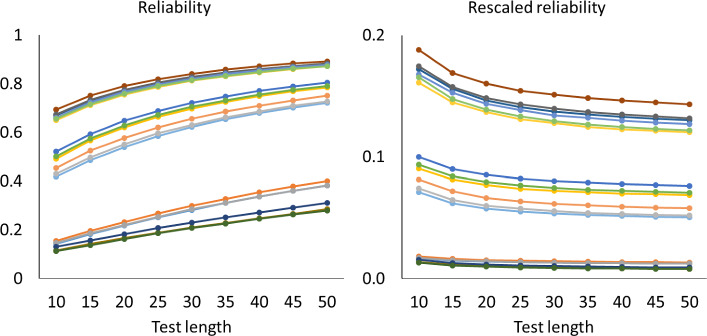
Mean reliabilities in Simulation Study 1 for five-dimensional cases with $a_{\textrm{max}}=2$. Note. Mean reliability and mean rescaled reliability as a function of test length, in 18 cases of five-dimensional models. The cases are represented by different colors. Each point is based on 1000 random test versions.

**Fig. 4:**
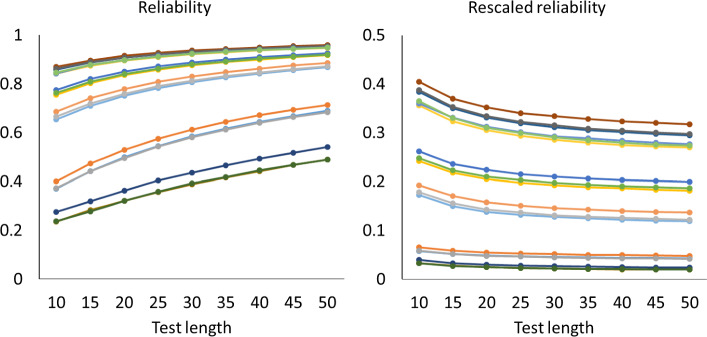
Mean reliabilities in Simulation Study 1 for five-dimensional cases with $a_{\textrm{max}}=5$. Note. Mean reliability and mean rescaled reliability as a function of test length, in 18 cases of five-dimensional models. The cases are represented by different colors. Each point is based on 1000 random test versions.

Figures 1, 2 and 3 show how the mean of the reliabilities $\rho (\textbf{S}_{n}$) and mean of the rescaled reliabilities $SB\! \left( \rho (\textbf{S}_{n}),\frac{1}{n} \right)$ depend on the test length in all cases with $a_{\textrm{max}}=2$. The left-hand panels show how the mean reliabilities increase with test length: They follow largely the pattern expected if the Spearman–Brown formula holds. The right-hand panels give a more detailed account via the mean rescaled reliabilities, which should stabilize according to theorem 1. For the one-dimensional tests in Fig. 1, the approximately horizontal lines suggest that the mean rescaled reliabilities are indeed stable. A Friedman rank test revealed that differences were significant in 10 out of 18 cases, but the effects were small. Across all 18 cases, the largest absolute deviation between means of rescaled reliabilities from the same case with different test lengths was 0.0098, which occurred when the mean rescaled reliability equaled 0.38. For the two-dimensional tests of Fig. 2, however, the mean rescaled reliabilities decreased gradually for the first 20 to 30 items, which implies that these reliabilities have a positive bias for short tests. For the five-dimensional tests of Fig. 3 the positive bias was larger. The rescaled reliabilities are also lower here than in the unidimensional cases, and one may wonder whether the rescaled lower reliabilities cause the bias. However, Fig. 4 shows the rescaled reliabilities in the five-dimensional cases with $a_{\textrm{max}}=5$; they have about the same magnitude as in the unidimensional cases with $a_{\textrm{max}}=2$, and yet there is a clear bias in the five-dimensional case and not in the unidimensional case. The other results with $a_{\textrm{max}}=5$ are essentially the same as with $a_{\textrm{max}}=2$ and are therefore not displayed.

A related question is whether the reliability with a given test length can predict the reliability at another test length. In this setting, lengthening and shortening of the test are associated with different knowledge states. If the investigator starts with a long test of which the reliability is known, then the item parameters can usually also be estimated and used to calculate what the reliability of any shortened version is (e.g., Raborn et al., 2020). For example, suppose the test constructor starts with 100 items, numbered 1–100 and she estimates the reliability with McDonald’s $\omega _{t}$, and wants to know what the reliability will become if only the items with numbers 40–60 are used. McDonald’s $\omega _{t}$ is computed from the factor loadings and unicities, so the test constructor apparently has estimates of the factor loadings and unicities of all items 1–100, and therefore she can compute the value of $\omega _{t}$ for items 40–60 directly from the available data, without using the Spearman–Brown formula. She might still apply the Spearman–Brown formula, but this seems futile because she already has a better answer. On the other hand, if the investigator starts with a short test, the additional items often not exist yet, and the Spearman–Brown formula may yield an incorrect prediction. In this setting, shortening the test requires in-sample prediction, whereas lengthening the test requires out-of-sample prediction.

**Fig. 5:**
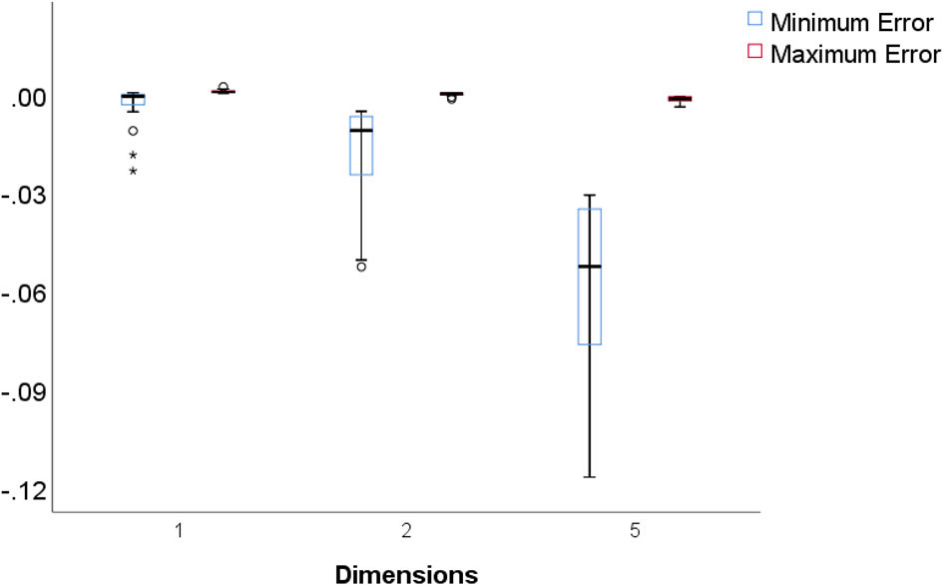
Boxplots of the minimum and maximum errors in prediction of mean reliabilities. Note. Each boxplot is based on 18 minima and maxima, corresponding to 18 cases with $a_{\textrm{max}}=2$. Each minimum or maximum is based on 36 predictions of a mean reliability of one test length from a mean reliability of another test length, for 9 different test lengths.

For each of the mean reliabilities shown in Figs. 1, 2, and 3, we computed how well it can be predicted out-of-sample from the mean reliabilities with smaller test length, and how well it can be predicted in-sample from the mean reliabilities with larger test lengths The reliability of one random test form of *n* items is $\rho (\textbf{S}_{n}$) (computed with numerical integration from the 2PL model), and for each parameter case we generated 1000 random test forms; let $\bar{\rho _{n}}$ be the mean of $\rho (\textbf{S}_{n}$) over these 1000 test forms. If the mean reliability of tests of length *n* is predicted from the mean reliability of tests of length *m*, then the observed value of the mean reliability is $\bar{\rho _{n}}$, the predicted mean reliability is $SB\! \left( \bar{\rho _{m}},\frac{n}{m} \right)$, and the error is $\bar{\rho _{n}}- SB\! \left( \bar{\rho _{m}},\frac{n}{m} \right)$. In some cases we consider the rescaled reliability, and then the rescaled mean reliability is $SB\! \left( \bar{\rho _{n}},\frac{1}{n} \right)$ and the predicted rescaled mean reliability is $SB\! \left( \bar{\rho _{m}},\frac{1}{m} \right)$. The following patterns can be expected. We noted already that in Figs. 1, 2, and 3, the rescaled reliabilities tend to decrease with test length, especially in the multidimensional cases, and we described this as a positive bias of short tests. Since the function $SB\! \left( \rho ,n \right)$ is increasing in $\rho$, this implies that out-of-sample predictions, relevant in test lengthening, have a positive bias too, especially in the multidimensional cases; that is, the observed reliability of the lengthened test tends to be less than the predicted reliability in multidimensional cases. Conversely, for test shortening, the observed reliability tends to be greater than the predicted reliability in multidimensional cases. For unidimensional cases, Figs. 1, 2 and 3 show that the rescaled reliabilities are approximately stable with test length, and therefore both prediction errors will be small in these cases. These expected patterns are confirmed in Fig. 5, which shows boxplots of the minimum and maximum errors in test lengthening as a function of the number of dimensions. The figure shows that the size of prediction errors depends strongly on the number of dimensions. The out-of-sample errors are between 0.00 and $-0.02$ in the unidimensional cases, whereas they were between 0.0 and $-0.12$ in the five-dimensional cases. The prediction errors in test shortening, which are not displayed in Fig. 5, have the opposite direction, as expected: between $-$0.01 and 0.02 for unidimensional cases and between 0.00 and 0.08 in the five-dimensional cases.

We used a two-factor ANOVA for estimating the explained variances. For test shortening, the number of dimensions explained 84% of the variance in maximum absolute errors, while the mean discrimination parameter and its interaction with the number of dimensions explained 2% and 11%, respectively. Together these two factors explained 98% of the variance in maximum absolute errors. For test lengthening these percentages were 62% (number of dimensions), 22% (mean discrimination parameter), 8% (interaction), and 92% (together). In the unidimensional cases the largest maximum absolute errors occurred when the discrimination parameters had a low mean (0.17) with a J-shaped distribution. In the other unidimensional cases the maximum absolute errors were at most 0.006, both in shortening and lengthening

We reject the hypothesis that the Spearman–Brown formula generally yields accurate predictions for short multidimensional tests, but we cannot yet reject the hypothesis that it yields accurate predictions for short unidimensional tests. Therefore, we focus on unidimensional tests in the second simulation study and put this hypothesis to the test.

### Simulation Study 2: Unidimensional With Binary or Irregular Item Parameters

In this section, we test whether the Spearman–Brown formula yields accurate predictions of mean reliability in unidimensional tests under the 2PL model. In the previous section, the item parameters were drawn from beta-distributions, but in the present section, we use more irregular distributions that may reveal violations of the hypothesis. We studied the following distribution types.

1. Binary item parameters. To create extreme situations, we generated cases with $a_{\omega }\in \{0.5, 2.0\}$ and $b_{\omega }\in \{-1.7, 1.7\}$. The probabilities of $a_{\omega }=0.5$ were 0.1, 0.3, 0.5, 0.7, and 0.9, and the probabilities of $b_{\omega }=-1.7$ were 0.1, 0.5, and 0.9. This created 15 parameter cases (5 values of $P(a_{\omega }=0.5$) by 3 values of $P(b_{\omega }=-1.7)$). For each parameter case, we created an item pool of 1000 items.
2. As point 1 but with $b_{\omega }\in \{0, 1.7\}$
3. Irregular distributions of $a_{\omega }$ and $b_{\omega }$. This was done by creating 100 cases of small item pools of 10 items each, with of $a_{\omega }$ and $b_{\omega }$ drawn from uniform distributions on [0.5, 2.0] and $[-2.0, 2.0]$, respectively. In each item pool the distribution was irregular because of the small pool size. Moreover, the two item parameters can be correlated within a small item pool.
4. Reported item parameters from the literature, two parameter cases:
  - Hays et al. (2000, their Table 4: 11 items) and
  - Pedraza et al.(2011, their Table 2: 60 items);

In each parameter case, 1000 random test versions of 50 items were created, and the reliabilities were computed starting with a random 10-item test and adding batches of 5 random items, thus creating additional test lengths of 15, 20, 25, 30, 35, 40, 45, and 50 items. We considered test shortening by deleting batches of 5 items starting at 50 items. The maximum error of prediction, where mean reliabilities associated with different test lengths were predicted from each other, was computed for test shortening and lengthening situations separately. The rescaled mean reliabilities $SB\! \left( \bar{\rho _{n}},\frac{1}{n} \right)$ were computed for each parameter case and each test length, and the maximum absolute difference between the values of $SB\! \left( \bar{\rho _{n}}, \frac{1}{n} \right)$ was computed in each parameter case. These are then summarized per distribution type (points 1 through 4b above).

Table 1 provides the results. For the prediction of mean reliabilities, the maximum error from all cases was 0.012. For the rescaled mean reliabilities, the maximum error from all cases was 0.017. These error margins are acceptable.

**Table 1:** Maximum Errors of Simulation Study 2.

Distribution type	Maximum error in in-sample or out-of-sample prediction of mean reliabilities	Maximum absolute difference of rescaled mean reliabilities
1	0.012	0.017
2	0.005	0.009
3	0.006	0.013
4a	0.002	0.003
4b	0.0004	0.003

### Simulation Study 3: Standard Deviations of the Reliabilities

**Fig. 6:**
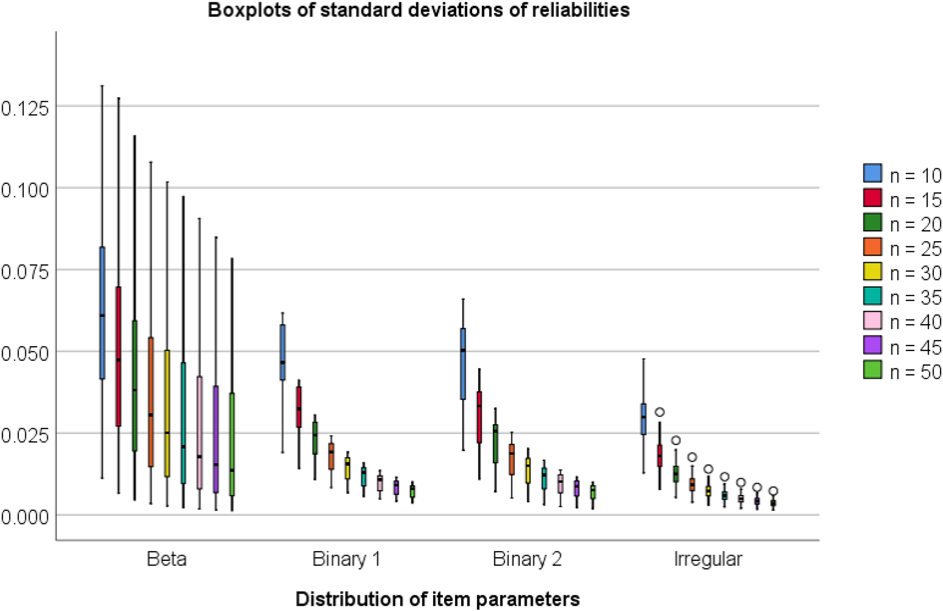
Boxplots of the standard deviations of the reliabilities as a function of the distribution type of the parameters and the length of the test versions. Note. Only unidimensional items with $a_{\textrm{max}}=2$ were used in this plot. Each boxplot is based on 54 (Beta) or 15 (Binary 1 and Binary 2) or 100 (Irregular) standard deviations, and each standard deviation is based on 1000 reliabilities of random test forms drawn from the same item pool.

The previous sections considered how well mean reliabilities can be predicted from each other. In practical situations, however, the test constructor does not have several test versions but rather a single test version. Even if the test constructor knows the correct reliability of this test version based on a large subject sample, the test version’s reliability might be different from the mean reliability of all test versions of the same length. To get an impression of the magnitude of this variation, we computed the standard deviation of the reliabilities in each unidimensional case and each test length used in the previous sections. Figure 6 shows boxplots of the standard deviations. As we expected, the standard deviations tend to decrease within each distribution type as test length increases. Aggregated over all case, the medians of the standard deviations decrease from 0.034 with $n=10$ to 0.004 with $n=50$. The largest standard deviation was 0.131. The 90^th^ percentile of the standard deviations decreased from 0.074 for $n=10$ to 0.031 for $n=50$.

Finally, we also computed in the 18,000 item pools of 2PL parameter cases the correlation between the observed reliability with 50 items, $\rho (\textbf{S}_{50}$), and the predicted reliability for 50 items based on the reliability with 20 items, $SB\! \left( \rho \! \left( \textbf{S}_{20} \right) ,2.5 \right)$. The observed reliabilities have a mean of 0.86 with standard deviation 0.14; the correlation was 0.96, with a mean absolute error of 0.02. The 90^th^ percentile of the absolute error was 0.06. The maximum absolute error was 0.43, so the predictions are not infallible. When predicting from 10 items with $SB\! \left( \rho \! \left( \textbf{S}_{10} \right) ,5 \right)$, the correlation is 0.91, and we consider these predictions as an educated guess.

## Discussion

We showed that in a one-facet universe with randomly sampled items with uncorrelated error-scores and finite second moments, as the number of items increases, the reliability of the total score approaches 1 at the rate of the Spearman–Brown formula. This result holds regardless of the dimensionality of the items. That the reliability usually converges to 1 was presumed widely but never proven; we have now produced a rigorous proof. That the convergence rate is given by the Spearman–Brown formula is more surprising, because it is generally believed that this formula requires parallel items or test parts. Our result shows that the Spearman–Brown formula is asymptotically correct for randomly sampled items that are not parallel and not even unidimensional.

We have investigated to which extent the Spearman–Brown formula can be used to predict test-score reliability resulting from changing test length for nonparallel items. For short tests simulations where random test versions of items with known item parameters of a 2PL model were drawn and the IRT model-based reliabilities were computed with numerical integration, showing that reliability had a substantial positive bias for multidimensional item sets. For unidimensional items reliability was almost unbiased, and the mean reliabilities for different test lengths could be predicted from each other using the Spearman–Brown formula. However, a single short test version can have a reliability that deviates substantially from the mean reliability of test versions of the same length from the same item pool, although most cases that were considered had standard deviation smaller than 0.034 even with $n=10$.

Whether the accuracy of the Spearman–Brown formula depends on the dimensionality of the items or that random sampling of items is sufficient, depends on the test length. The dimensionality does not matter if the test is sufficiently long, as Theorem 1 established, but for short tests dimensionality is important, as the simulation studies demonstrated.

The conclusion is that the Spearman–Brown formula can reasonably be used to predict reliability after changing the test length with randomly sampled items, provided that the initial test is long or the test items are 2PL-unidimensional. In test lengthening the reliability of the original test may deviate substantially from the mean reliability, contaminating the prediction. However, for 2PL-unidimensional item pools bias was negligible (despite being significant), and therefore prediction with the Spearman–Brown formula can be viewed as an educated guess, even though reliability cannot be predicted with certainty as would be the case with parallel items, an unrealistic situation for sure. For multidimensional item pools positive bias of the reliability of short tests was non-negligible. Consequently test constructors who use the Spearman–Brown formula in multidimensional cases to predict reliability of a longer test will easily be too optimistic.

### Is Random Selection of Items Realistic?

There are several examples where random selection of items is possible. First, we point out that the concept of an “item” here is no more than a component of measurement, and therefore, in addition to being a problem or a question as we are used to it may correspond to a time point randomly selected from an interval, or a rater randomly selected from a population of raters. In the latter examples, increasing the test length then means increasing the number of time points or increasing the number of raters. Obviously, random selection is possible in these cases, and our result applies to it. For example, in the experience sampling method (ESM) subjects are asked to answer a short questionnaire about their current mood or thoughts on multiple occasions per day. van Lankveld et al. (2018, 2021) and van Tuijl et al. (2022) used this method with mood scales of intimacy and sexual desire administered on 10 moments per day for seven consecutive days. Subjects wore a wristwatch that prompted them with beeps to fill in the questionnaire. The beeps were “quasi-randomly” distributed around time points separated by 90 min each, between 7:30 AM and 10:30 PM. Although the time points are a stratified random sample rather than a simple random sample, this additional control is likely to reduce the error variance and accelerate convergence of the reliability. In this research method, the subject means over time are not the primary focus of interest, but they are used to compute “person-centered” data, and their reliability is therefore important and reported (Van Lankveld et al., 2021, pp. 316–317).

If we confine the concept of an item to a test question, there are still examples of random item selection. In Supplementary Material we show a webapp that generates statistics questions about graphs of univariate or bivariate distributions. A test item here consists of a verbal question (like “the correlation is greater at …”) and two graphs. The graphs are randomly generated with continuous distributions, and therefore the app can generate infinitely many items. The user is presented a random sequence of items whenever they start the app. The app evaluates the answer of the student and keeps the score. Similar apps have been used in large-scale examinations by one of the authors.

There are several neuropsychological tests where the items can be thought of as drawn from an infinite item pool. In the Eriksen flanker task (Eriksen and Eriksen, 1974), subjects are presented letter strings (SSSSS, SSHSS, HHSHH, HHHHH) and are instructed to press a button with one hand if the central letter is an H and with the other hand if the central letter is an S. On trials where participants respond incorrectly, specific event-related potentials (ERPs) are measured from the scalp with the use of electro-encephalography (EEG). The ERPs are averaged over error trials, and their mean is used as the test score. There are several other neuropsychological tests with the same design, such as the Stroop test. The number of trials is limited only by the will of the subject and the researcher. The items here are only the trials on which the subject makes an error (which provokes interesting neurological responses; correct trials lack such a response), which is a sample from the total number of trials. One can debate whether this is truly a random process, because the error making is governed by internal processes of the subject, but at least one group of authors advocates to treat this as a random factor (Clayson et al., 2021).

An example in the domain of cognitive tests is mental rotation tasks, where subjects are presented with two or more pictures of block figures that may or may not be a 3-dimensional rotation of each other, and are asked which figures, if any, have the same shape. With a typical number of blocks, like 11, the number of shapes is finite but large, and the number of rotations is infinite, thus defining an infinite item pool of which a random sample can be generated with an algorithm. Even if test constructors did not literally draw items from a pool, it is hard to see why many other items would not be equally adequate, and random sampling seems a reasonable model for this.

Note that for random selection of items it is not enough to have infinitely many items; their order must be randomized too in the drawing process, but not necessarily in the presentation to the subject. For example, consider the set of items of the form “$2\times n= ?$” with $n\in \mathbb {N}$. Two examples are “$2 \times 17 =$ ?” and “$2 \times 212 =$ ?” If a test of 10 items is created and one uses $n=1,...,10$, that would not be a random sample because it contains only the easiest items. Instead, a probability distribution over $\mathbb {N}$ should be used, for example the geometric distribution with $\pi =0.02.$ The random items numbers could then be, for example, 7, 30, 217, 1, 4, 8, 161, 33, 137, 24. Once it is determined that only these items will be presented, they can be arranged in a different order for presentation to the subjects. This example illustrates that random selection of items does not mean that all items of the pool should have the same probability of being drawn. In fact, with a countable infinite pool it is impossible that all items have the same probability, which is a well-known fact in probability theory. Thus, a higher concentration of some kind of items does not refute random selection of items.

For most psychological tests it is true that, although the items are not literally randomly drawn from a larger pool, many similar items are conceivable. We contend that this may be modelled as random drawing of items, provided that there is no systematic drift in item parameters.

## Consequences For Practitioners

Until now, only if items were parallel, a condition impossible to satisfy with real items was the user certain that test lengthening yielded a higher reliability, but in practice, (s)he had to rely on experience and intuition for expecting this effect. With our result, the user now knows that adding items, also if they are not parallel, eventually increases reliability. The importance may escape the user and the reader as well, but this would mostly be because decades of test construction have fostered the belief that adding items has the expected effect, but without proof. We provide this proof now and theoretically justify the wisdom test constructors practiced for decades. Here are some specifics.

1. In domains where test constructors can generate arbitrarily many items in a stable manner, they can take the test reliability as close to 1 as they wish by adding enough items. This is true even if the test is multidimensional. Note that different kinds of reliability pertain to different kinds of items: If the items are questions from a large question pool, add questions from the same pool; if the items are time points in a time interval, add time points from the same interval; if the items are trained raters from a certain population, add trained raters from the same population.
2. A limited reliability therefore often reflects the decision of the test constructor to stop generating items rather than a fundamental property of the domain. For example, personality tests usually have a reliability coefficient of about 0.80, whereas intelligence tests usually have a reliability coefficient of about 0.95. These values are not properties inherent to the domains of personality and intelligence; they rather reflect decisions of test constructors to stop adding items.
3. Conversely, a high reliability does not say anything about the quality of the items; it may just reflect that the test contains a large number of items. Similar remarks have been made with respect to coefficient $\alpha$ (Sijtsma, 2009). However, in the context of $\alpha$ there is often a discussion whether it is a sound estimate of reliability. In this article we showed that even if we have the “true” reliabilities, they go to 1 if we continue adding items from the same pool.
4. If you want to know how much the reliability will improve with a given lengthening factor for unidimensional items, then the Spearman–Brown formula gives a reasonable estimate. It is well-known that the estimate is exactly correct only for parallel items, but for randomly selected items from a unidimensional pool the Spearman–Brown formula still describes correctly how the expected value of the reliability increases with test length.
5. This result with respect to the Spearman–Brown formula is also true for long tests with items that are randomly selected from multidimensional pools.

## Data Availability

The simulated data of Figs. 1, 2, 3, 4, 5, and 6 and Table 1, and the code that generated it, are available in the Open Science Framework repository at https://osf.io/v7k94/?view_only=b724b2f74c9949828f52154c7e06a6eb

## Appendix

This appendix contains the proof of the theorem in the main text. A lemma is first stated and proved, and after that the theorem is stated and proved. Recall that the section “Notation and general assumptions” described assumptions that hold throughout this article (randomly selected items, uncorrelated error-score variables, finite second moments). The definition of $\mathcal {F}$ needs further attention. Let $(A, \mathcal {A}, P$) be the probability space on which the observed-score variables $\{X_{\omega }\vert \,\omega \in \Omega \}$ are defined. We assume that $\mathcal {F}\subseteq \mathcal {A}$ is a $\sigma$-field. In the main text we described $\mathcal {F}$ as a collection of variables, which is consistent with the notational conventions of conditional expectations to replace the sigma field generated by conditioning variables by the variables themselves. More precisely however, $\mathcal {F}$ is a sigma field that is contained in $\mathcal {A}$. No other direct assumptions about $\mathcal {F}$ are needed. However, some choices of $\mathcal {F}$ may lead to violation of other assumptions, such as uncorrelated error variables, and such $\mathcal {F}$s are excluded.

### Proposition 1

If the error-score variables are uncorrelated, then $\mathrm{var\!} \left( E_{R_{1}}+E_{R_{2}}\vert (R_{1},R_{2}) \right) =\mathrm{var\!} \left( E_{R_{1}}\vert (R_{1},R_{2}) \right) +\mathrm{var\!} \left( E_{R_{2}}\vert (R_{1},R_{2}) \right)$

### Proof of Proposition 1

Recall that $(R_{1},R_{2}$) just fixates the first two items, and there is probability 0 that these two items are equal. For any two items $\omega , \upsilon \in \Omega , \omega \ne \upsilon$, we have

$$\begin{aligned} \mathrm{var\!}\left( E_{R_{1}}+E_{R_{2}}\vert \left( R_{1},R_{2} \right) =(\omega ,\upsilon ) \right) =\mathrm{var\!} \left( E_{\omega }+E_{\upsilon }\vert \left( R_{1},R_{2} \right) =(\omega ,\upsilon ) \right) \end{aligned}$$

It was assumed that the $R_{1}, R_{2},...$ are independent of $\{E_{\omega }\vert \omega \in \Omega \}$, therefore

$$\begin{aligned} =\mathrm{var\!}\left( E_{\omega }+E_{\upsilon } \right) \end{aligned}$$

and because of the uncorrelated errors we have

$$\begin{aligned} =\mathrm{var\!}\left( E_{\omega } \right) +\mathrm{var\!} \left( E_{\upsilon }\right) \end{aligned}$$

and again using that the $R_{1}, R_{2},...$ are independent of $\{E_{\omega }\vert \omega \in \Omega \}$

$$\begin{aligned}= & {} \mathrm{var\!}\left( E_{\omega }\vert \left( R_{1},R_{2} \right) =(\omega ,\upsilon ) \right) +\mathrm{var\!}\left( E_{\upsilon } \vert \left( R_{1},R_{2}\right) =(\omega ,\upsilon ) \right) \\= & {} \mathrm{var\!}\left( E_{R_{1}}\vert (R_{1},R_{2})=(\omega ,\upsilon )\right) +\mathrm{var\!}\left( E_{R_{2}}\vert (R_{1},R_{2})=(\omega ,\upsilon )\right) \end{aligned}$$

$$\square $$

### Proposition 2

If the error-score variables are uncorrelated, then

$$\begin{aligned} \mathrm{var\!}\left( E_{\textbf{S}_{n}}\vert \textbf{S}_{n}\right) =\sum \limits _{i=1}^n \frac{\mathrm{var\!}\left( E_{R_{i}}\vert \textbf{S}_{n} \right) }{n^{2}} \end{aligned}$$

### Proof of Proposition 2

Similar to the proof of Proposition 1, but with more variables. $\square$

Let

$$\begin{aligned} \bar{\varepsilon ^{2}}(\textbf{S}_{n}) =\sum \limits _{i=1}^n {\varepsilon ^{2}\! \left( R_{i} \right) } /n \end{aligned}$$

This is a mean of *n* i.i.d. variables $\varepsilon ^{2}(R_{i}).$

### Lemma 1

If the error-score variables are uncorrelated, then $\mathrm{var\!}\left( E_{\textbf{S}_{n}}\vert \textbf{S}_{n} \right) =\bar{\varepsilon ^{2}}(\textbf{S}_{n})/n$ and $\mathbb {E}\!\left( \bar{\varepsilon ^{2}}\!\left( \textbf{S}_{n} \right) \right) =\mathbb {E}(\varepsilon ^{2}\!\left( R_{1} \right) )$

### Proof of Lemma 1

Using Proposition 2,

$$\begin{aligned} \mathrm{var\!}\left( E_{\textbf{S}_{n}}\vert \textbf{S}_{n}\right) =\sum \limits _{i=1}^n \frac{\mathrm{var\!}\left( E_{R_{i}} \vert \textbf{S}_{n} \right) }{n^{2}} \end{aligned}$$

The $R_{i}$ are independent, and therefore $\mathrm{var\!} \left( E_{R_{i}}\vert \textbf{S}_{n} \right) =\mathrm{var\!} \left( E_{R_{i}}\vert R_{i} \right) =\varepsilon ^{2}\! \left( R_{i} \right)$. This yields

$$\begin{aligned} =\sum \limits _{i=1}^n \frac{\varepsilon ^{2}\! \left( R_{i} \right) }{n^{2}} =\bar{\varepsilon ^{2}}(\textbf{S}_{n})/n \end{aligned}$$

Furthermore, since the $R_{i}$ are i.i.d, $\mathbb {E}(\varepsilon ^{2}\! \left( R_{i} \right) )=\mathbb {E}(\varepsilon ^{2}\! \left( R_{1} \right)$), and therefore

$$\begin{aligned} \mathbb {E}\left( \bar{\varepsilon ^{2}\!}\left( \textbf{S}_{n} \right) \right) =\mathbb {E}\left( \sum \limits _{i=1}^n {\varepsilon ^{2}\!\left( R_{i} \right) } /n \right) =\frac{\sum \limits _{i=1}^n {\mathbb {E}\left( \varepsilon ^{2}\! \left( R_{i} \right) \right) } }{n}=\mathbb {E}\left( \varepsilon ^{2}\! \left( R_{1}\right) \right) \end{aligned}$$

$$\square $$

### Theorem 1

Assume that the error-score variables are uncorrelated and that the true-score variables are bounded by some square integrable random variable $T_{\textrm{max}}$, that is, $\left| T_{\omega } \right| <T_{\textrm{max}}$ for all $\omega \in \Omega$ and $\mathbb {E}\left| T_{\textrm{max}} \right| ^{2}<\infty$. For the reliabilities $\rho (\textbf{S}_{n}$), as $n\rightarrow \infty$

$$\begin{aligned} SB\left( \rho \! (\textbf{S}_{n}),\frac{1}{n} \right) \rightarrow \frac{\mathrm{var\!}\left( T_{\infty } \right) }{\mathrm{var\!}\left( T_{\infty } \right) +E(\varepsilon ^{2}\!\left( R_{1} \right) )} \end{aligned}$$

with probability 1.

### Proof of theorem 1

By the definition of $\rho \left( \textbf{S}_{n} \right)$ and Lemma 1,

$$\begin{aligned} \rho \left( \textbf{S}_{n} \right) =\frac{\mathrm{var\!} \left( T_{\textbf{S}_{n}}\vert \textbf{S}_{n} \right) }{\mathrm{var\!} \left( T_{\textbf{S}_{n}}\vert \textbf{S}_{n} \right) +\bar{\varepsilon ^{2}}(\textbf{S}_{n})/n} \end{aligned}$$

which implies

$$\begin{aligned} SB\left( \rho (\textbf{S}_{n}),\frac{1}{n} \right) =\frac{\mathrm{var\!}\left( T_{\textbf{S}_{n}}\vert \textbf{S}_{n} \right) }{\mathrm{var\!} \left( T_{\textbf{S}_{n}}\vert \textbf{S}_{n} \right) +\bar{\varepsilon ^{2}}(\textbf{S}_{n})} \end{aligned}$$

Consider first convergence of the true-score variance. Note that the $T_{R_{i}}$ are exchangeable. Since it is assumed that $\left| \mathbb {E}\left( T_{R_{i}} \right) \right| <\infty$, one can define $T_{\infty }:=\mathbb {E}(T_{R_{1}}\vert \mathcal {F}$) and show that the $T_{R_{i}}$ are i.i.d. given $\mathcal {F}$ with lemma 4.1 of Dawid (1980). By the strong law of large numbers for conditional expectations (Majerek et al., 2005, theorem 4.2; Walk, 2008), $T_{\textbf{S}_{n}}\rightarrow T_{\infty }$ with probability 1.

Write $\textbf{S}_{\infty }=(R_{1},R_{2},...$). Since $\textbf{S}_{n}$ and $(R_{n+1},R_{n+2},...$) are independent, $\mathrm{var\!}\left( T_{\textbf{S}_{n}}\vert \textbf{S}_{n}\right) =\mathrm{var\!}\left( T_{\textbf{S}_{n}}\vert \textbf{S}_{\infty } \right)$. We have already established that $T_{\textbf{S}_{n}}\rightarrow T_{\infty }$ with probability 1, and therefore $\mathrm{var\!} \left( T_{\textbf{S}_{n}}\vert \textbf{S}_{\infty } \right) \rightarrow \mathrm{var\!}\left( T_{\infty }\vert \textbf{S}_{\infty } \right)$ with probability 1 by the dominated convergence theorem for conditional expectations (e.g., Billingsley, 1986, Th.34.2.v), using the hypothesis that the $\left| T_{\omega } \right|$ are dominated. However, $T_{\infty }$ is defined solely in terms of $\mathcal {F}$, which is independent of $\textbf{S}_{\infty }$; therefore $\mathrm{var\!} \left( T_{\infty }\vert \textbf{S}_{\infty } \right) =\mathrm{var\!} \left( T_{\infty } \right)$. In sum, $\mathrm{var\!} \left( T_{\textbf{S}_{n}}\vert \textbf{S}_{n} \right) \rightarrow \mathrm{var\!}\left( T_{\infty } \right)$.

Next, consider convergence of the error variance. By the strong law of large numbers (which may be applied because the $R_{i}$ are independent and $\textrm{var}(\varepsilon ^{2}\! \left( R_{i} \right) )<\infty$),

$$\begin{aligned} \bar{\varepsilon ^{2}}(\textbf{S}_{n}) \rightarrow \mathbb {E}(\varepsilon ^{2}\! \left( R_{1} \right) ) \end{aligned}$$

with probability 1, and therefore (note that it was assumed that $\varepsilon ^{2}\! \left( R_{1} \right) >0$)

$$\begin{aligned} SB\left( \rho (\textbf{S}_{n}),\frac{1}{n} \right) \rightarrow \frac{\mathrm{var\!}\left( T_{\infty } \right) }{\mathrm{var\!} \left( T_{\infty } \right) +\mathbb {E}(\varepsilon ^{2}\! \left( R_{1} \right) )} \end{aligned}$$

with probability 1. $$\square $$
